# Profiling COVID‐related experiences in the United States with the Epidemic‐Pandemic Impacts Inventory: Linkages to psychosocial functioning

**DOI:** 10.1002/brb3.2197

**Published:** 2021-07-03

**Authors:** Damion J. Grasso, Margaret J. Briggs‐Gowan, Alice S. Carter, Brandon L. Goldstein, Julian D. Ford

**Affiliations:** ^1^ Department of Psychiatry University of Connecticut School of Medicine Farmington CT USA; ^2^ Department of Psychology University of Massachusetts Boston Boston MA USA

**Keywords:** COVID‐19, disaster, epidemic, mental health, pandemic

## Abstract

The COVID‐19 pandemic has had a profound impact on the lives of individuals, families, and communities around the world with constraints on multiple aspects of daily life. The purpose of the present study was to identify specific profiles of pandemic‐related experiences and their relation to psychosocial functioning using the 92‐item Epidemic‐Pandemic Impacts Inventory (EPII). Data were collected as part of a cross‐sectional, online survey of adults (18+) residing in the Northeast region of the United States (*N* = 652) and recruited via online advertisements. Person‐centered latent class analysis (LCA) was applied to 38 pandemic‐related experiences that showed a significant bivariate correlation with perceived stress. Measures of psychosocial risk were also obtained. Results revealed five unique profiles of respondents based on patterns of pandemic‐related experiences. Three profiles representing about 64% of the sample were characterized by moderate to high exposure to adverse experiences during the pandemic and were more likely to screen positive for depression, anxiety, and posttraumatic stress. These profiles were differentiated by sociodemographic differences, including age, caregiving, and employment status. Two profiles differentiated by age and caregiver status represented about 36% of the sample and were characterized by relatively low exposure to adverse experiences and lower risk for psychosocial impairment. Findings support the EPII as an instrument for measuring tangible and meaningful experiences in the context of an unprecedented pandemic disaster. This research may serve to identify high‐risk subpopulations toward developing public health strategies for supporting families and communities in the context of public health emergencies such as the COVID‐19 pandemic.

## Graphical abstract

The Epidemic‐Pandemic Impacts Inventory (EPII) is a novel tool for measuring tangible epidemic or pandemic related experiences across several life domains. The current study administered the EPII along with psychosocial measures to a sample of 652 adults residing in the Northeast region of the United States during the height of the COVID‐19 pandemic. Applying person‐centered analytic methods to experiences captured on the EPII revealed unique subgroups of individuals with different constellations of pandemic‐related experiences and associated psychosocial impairment.

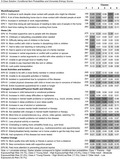

## 1 INTRODUCTION

In the first three‐month period, the COVID‐19 pandemic has had a profound impact on individuals, families, and communities across the world. Intensive health precautions have created constraints on mobility (e.g., sheltering in place, self‐quarantining), work and schooling (e.g., virtual commuting, homeschooling), family life (e.g., more intensive contact in primary household relationships, separation from extra‐household family members), and interpersonal relationships (e.g., social distancing, wearing masks). A number of survey studies have found evidence of an increase in self‐reported emotional and behavioral problems (e.g., anxiety, depression, stress disorders, insomnia) in the pandemic's immediate wake (Breslau et al., 2021; Fu et al., 2021; García‐Fernández et al., 2020; Murata et al., 2021; Petzold et al., 2020; Wu et al., 2020). However, few studies have examined the impact of specific pandemic‐related experiences or patterns of experiences, both negative and positive, on functioning, which is necessary for understanding the origins of burden on families toward developing public health interventions. Some exceptions include a study associating limitations in mobility with higher psychosocial distress in a United States sample (Devaraj & Patel, 2021) and an international study associating COVID exposure, government‐imposed quarantine level, and lifestyle changes with increased reports of depression and anxiety (Alzueta et al., 2021), as well as increases in domestic conflict with self‐reported sleep difficulties (Yuksel et al., 2021).

The goal of the present study was to employ a person‐centered analytic approach for empirically identifying specific profiles of pandemic‐related experiences and their relation to psychosocial functioning with information from the novel Epidemic‐Pandemic Impacts Inventory (EPII; Grasso et al., 2020). The EPII is a comprehensive, 92‐item inventory of experiences that extend across five thematic domains including adverse experiences specific to work/employment, home life, social activities and quarantine, and emotional/physical health and infection, as well as positive changes. The EPII is currently maintained in the National Institute of Health (NIH) Disaster Research Response (DR2) Repository of COVID‐19 Research Tools (https://dr2.nlm.nih.gov). Recent studies using the EPII have associated specific pandemic‐related experiences with increased risk for depression and anxiety (Alzueta et al., 2021; Yuksel et al., 2021), cumulative counts of adverse experiences with psychosocial distress and coping difficulties among teachers (Baker et al., 2021), and positive experiences with better psychosocial health in Scottish adults (Williams et al., 2021).

The present study sought to determine whether patterns of co‐occurring pandemic‐related experiences on the EPII define unique profiles of individuals that also differ on sociodemographic characteristics and psychosocial functioning. Profiles were empirically determined using latent class analysis (LCA), an exploratory, person‐centered, data‐driven approach for clustering individuals on a set of characteristics. LCA was applied to a subset of EPII items showing a significant correlation with a separate measure of perceived stress. Demonstrating unique profiles of pandemic‐related experiences that differentially predict psychosocial risk would support the construct validity of the EPII. Notably, traditional factor analytic methods grounded in classical test theory are not appropriate for evaluating the validity of instruments that inventory event‐type data (Felix et al., 2019), as is the case for the EPII. Classical methods treat items as indicators of latent constructs and assume an underlying, normally distributed latent variable or variables comprised of correlated indicators. LCA is not bound by these assumptions and provides the means to identify items or experiences that probabilistically co‐occur to characterize unique profiles or subgroups of individuals.

LCA also provides information beyond what is possible by summing event‐type data to create a cumulative count of experiences that may associate with risk. While this cumulative count approach is practical and statistically robust in predicting outcomes, drawbacks include erroneous assumptions that: (a) all items are equally associated with a particular outcome, (b) distances between sum scores are proportionately associated with an outcome, and (c) equivalent sum scores representing different combinations of items convey the same risk on an outcome (Netland, 2001). As such, the cumulative count approach, while informative, offers little to be learned about risk specific to individual exposures or unique constellations of co‐occurring exposures on outcomes.

In contrast, LCA uses maximum likelihood methods to empirically classify individuals into profiles or classes based on probabilistic patterns of co‐occurring exposures. LCA is not bound to linear assumptions and can be used to test the significance of different combinations of exposures on outcomes. The trauma exposure field has seen a burgeoning of studies using LCA to identify unique subgroups of individuals with different combinations of trauma exposures (Dierkhising et al., 2019; Ford et al., 2013; Goldstein et al., 2020; Grasso et al., 2013; Grasso, Dierkhising, et al., 2016; Grasso, Petitclerc, et al., 2016). Additionally, to our knowledge, only one study has applied LCA to disaster‐specific experiences to examine the impact of flooding on families (Felix et al., 2019). The latter study identified four unique profiles that were differentially associated with depression, anxiety, and PTSD symptoms.

To this end, the current study applied LCA to stress‐related pandemic experiences assessed with the EPII in a cross‐sectional survey conducted in the Northeast region of the U.S, the location of the initial epicenter of the pandemic in the U.S. The first aim was to use exploratory, person‐centered LCA to examine whether unique profiles of individuals could be identified based on different patterns of probabilistically co‐occurring stress‐related pandemic experiences endorsed on the EPII. A second aim examined whether identified profiles of individuals would significantly differ on sociodemographic characteristics and psychosocial indicators. Identifying unique profiles of individuals with distinct patterns of pandemic‐related experiences that differentially associate with psychosocial risk would support the validity of the EPII as an inventory of experiences relevant to understanding the impact of the pandemic on daily life, health, and well‐being. The availability and efficient and validated measure of the specific impacts of mass disasters is critical for both current and future prevention and intervention efforts.

## 2 METHOD

### 2.1 Procedures

An anonymous online survey using Qualtrics Survey Software was deployed via advertisements posted on social media (Facebook, Twitter, Instagram, Reddit), listservs, and ResearchMatch.org to recruit a convenience sample of adults residing in the Northeast region of the U.S. The survey was comprised of measures obtaining: (a) sociodemographic characteristics, (b) negative and positive pandemic‐related experiences on the EPII, (c) perceived stress, (d) symptoms of depression and anxiety, (e) PTSD symptoms, and (f) perceived social support. Completion of the survey implied consent. Upon completion, participants could opt to enter a lottery to receive a $25 electronic gift card (1/100 draw).

Four quality checks were implemented throughout the survey in the form of multiple‐choice questions in which the correct answer was embedded in the question. Incorrectly answering the first quality check resulted in a warning that the survey would be discontinued if any one of the subsequent quality checks were incorrectly answered. Forced response prevented missing data. For each item, participants had the option of selecting “*I choose not to respond*,” which happened infrequently. When this occurred for measures of psychological constructs, items were imputed with the average of items within their respective scale. The average number of non‐disclosed items for each measure was <0.25%. The study protocol was reviewed by the University of Connecticut School of Medicine Human Subjects Review Board and deemed exempt. Research data are not shared.

### 2.2 Sample characteristics

The survey documented a total of 853 responses over a 4‐week period. Among the 853 responses, 201 (23.6%) failed the quality check forcing the survey to discontinue. This resulted in an analytic sample of 652 (76.4%). Table 1 presents sociodemographic characteristics. The majority of the sample resided in Connecticut (45.1%), where the survey originated. Respondent age ranged from 18 to 85 (*M* = 47.01, *SD* = 14.36). Most of the sample self‐identified as female (83%), White (89.1%), and Non‐Hispanic/Latinx (94.8%). Eight percent reported an annual household income <$20,000 and 16.5% reported no insurance or receiving Medicaid/Medicare. The majority of the sample reported owning or renting their home (88.2%), having earned a bachelor's degree or higher (75.8%), and being in a long‐term relationship (71.5%). Most of the sample reported being currently employed (61.3%). Students comprised 9.4% of the sample.

**TABLE 1:** Sociodemographic Characteristics

Variable	*n*	%
State		
Connecticut	294	45.1
Maine	16	2.5
Massachusetts	88	13.5
New Hampshire	18	2.8
New Jersey	50	7.7
New York	132	20.2
Pennsylvania	36	5.5
Rhode Island	9	1.4
Vermont	9	1.4
Age		
18–29	103	15.8
30–39	99	15.2
40–49	152	23.3
50–59	154	23.6
60–69	108	16.6
70–79	34	5.2
80–89	2	0.2
Gender		
Female	541	83.0
Male	98	15.0
Non‐binary	9	1.4
Non‐disclosed	4	0.6
Ethnicity		
Hispanic/Latinx	34	5.2
Race		
African American	24	3.7
American Indian or Alaska Native	1	0.2
Asian	18	2.8
Native Hawaiian or Pacific Islander	1	0.2
White	581	89.1
Biracial	14	2.1
Non‐disclosed	13	2.0
Education		
High School	23	3.5
Vocational/Trade	8	1.2
Some College	81	12.4
Associates Degree	45	6.9
Bachelor's Degree	212	32.5
Masters	202	31.0
Doctoral or Advanced	80	12.3
Non‐disclosed	1	0.2
Insurance		
No Insurance	8	1.2
Medicaid/Medicare	100	15.3
Commercial	520	79.8
Non‐disclosed	24	3.7
Annual household income		
Less than $10,000	19	2.9
$10,000–$19,999	33	5.1
$20,000–$29,999	26	4
$30,000–$39,999	31	4.8
$40,000–$49,999	26	4
$50,000–$59,999	39	6
$60,000–$69,999	29	4.4
$70,000–$79,999	50	7.7
$80,000–$89,999	35	5.4
$90,000–$99,999	34	5.2
$100,000–$149,999	134	20.6
More than $150,000	138	21.2
Non‐disclosed	58	8.9
Relationship status		
Long‐term relationship	466	71.5
Single	173	26.5
Non‐disclosed	13	2.0
Living arrangement		
Own/rent	575	88.2
Parent/guardian's home	57	8.7
Son or daughter's home	5	0.8
Homeless shelter	5	0.8
Friend or relative's home	7	1.1
Non‐disclosed	3	0.5
Lives alone	80	12.3
Young child (≤12)		
Lives with me	104	16.0
Lives elsewhere	1	0.2
No	547	83.9
Older child/adolescent (13–17)		
Lives with me	101	15.5
Lives elsewhere	9	1.4
No	542	83.1
Child older than 18		
Lives with me	114	17.5
Lives elsewhere	155	23.8
No	383	58.7
Older adult		
Adult > 60 years in home	207	31.7
Employment status		
Full‐time	400	61.3
Part‐time	66	10.1
Unemployed, laid off, furloughed	107	16.4
Retired	68	10.4
Non‐disclosed	11	1.7
Student status		
Full‐time	40	6.1
Part‐time	21	3.2
Non‐student	556	85.3
Non‐disclosed	35	5.4

### 2.3 Measures

#### 2.3.1 The Epidemic‐Pandemic Impacts Inventory (Grasso et al., 2020)

The Epidemic‐Pandemic Impacts Inventory (EPII) is a 92‐item inventory of pandemic‐related experiences across several life domains: Work Life (12‐items), Home Life (19 items), Social Activities and Isolation (18‐items), Emotional/Physical Health and infection (24‐items), and Positive Change (19‐item). All domains except for the Positive Change domain index negative or adverse experiences. Each item has a response set of “*Yes, Me*”, “*Yes, Person in Home*”, “*No*”, and “*Not Applicable*”, except for items 42, 43, and 65, which pertain to the household more globally. The first two responses can be mutually inclusive. The second response (“*Yes, Person in Home*”) can pertain to family or non‐family living in the home and is conceptualized as having a potential impact on the respondent. For the purposes of this paper, the two “*Yes*” responses were collapsed, as were the “*No*” and “*N/A*” responses, which resulted in dichotomous indicators.

#### 2.3.2 The Perceived Stress Scale (Cohen et al., 1983)

The Perceived Stress Scale (PSS) is a 10‐item measure of one's perception of life is unpredictable, uncontrollable, and overloaded (0 = “*Never*,” 1 = “*Almost Never*,” 2 = “*Sometimes*,” 3 = “*Fairly Often*,” 4 = “*Very Often*”). The total score is the sum of all items (*α* = .80).

#### 2.3.3 The Patient Health Questionnaire‐9 (Kroenke et al., 2001)

The Patient Health Questionnaire‐9 (PHQ‐9) is a 9‐item self‐report measure of depressive symptoms over the past two weeks that range from 0 (“*Not at All*”) to 3 (“*Nearly Every Day*”). Total score ranges from 0 to 27. It has established construct validity and excellent test‐retest reliability (*r* = .84; Kroenke et al., 2001). In the present study, internal consistency was .88. The average number of non‐disclosed/imputed items across participants was 0.15%.

#### 2.3.4 The Generalized Anxiety Disorder‐7 (Spitzer et al., 2006)

The Generalized Anxiety Disorder‐7 (GAD‐7) is a 7‐item self‐report measure of generalized anxiety disorder symptoms over the past two weeks that range from 0 (“*Not at All*”) to 3 (“*Nearly Every Day*”). Total score ranges from 0 to 21. It has good convergent validity with other anxiety scales and excellent test‐retest reliability (intra‐class correlation = .83; Spitzer et al., 2006). In the present study, the internal consistency was .92. The average number of non‐disclosed/imputed items across participants was 0.22%.

#### 2.3.5 The Primary Care PTSD Screen for DSM‐5 (Prins et al., 2016)

The Primary Care PTSD Screen for DSM‐5 (PC‐PTSD‐5) is a self‐report measure of DSM‐5 defined PTSD symptoms. Five Yes/No items assess symptoms yielding a continuous symptom score ranging from 0 to 5. Previous research has demonstrated that the PC‐PTSD‐5 predicts PTSD diagnosis with a high degree of accuracy and has good test‐retest reliability (Prins et al., 2016). In the present study, internal consistency was .75. The average number of non‐disclosed/imputed items across participants was 0.01%.

#### 2.3.6 The Duke‐UNC Social Support Questionnaire (Broadhead et al., 1988)

The Duke‐UNC Social Support Questionnaire is a 5‐item self‐report measure assessing one's perception of the availability of support or assistance to fulfill needs. Each item assesses the degree/quantity to which a person feels that they have access to different indicators of social support using a 5‐point Likert scale ranging from 0 (“*None of the Time*”) to 5 (“*All of the Time*”). The Social Support Questionnaire has convergent validity with other measures of social support and general health and good two‐week test‐retest reliability (*r* = .66; Broadhead et al., 1988). In the present study, the internal consistency was .88. The average number of non‐disclosed/imputed items across participants was 0.15%.

### 2.4 Analytic approach

Descriptive statistics were calculated with Mathworks Inc. Matlab software (2020a). Distributional properties of dependent variables were examined for non‐normality and all measures fell within the acceptable range for skewness and kurtosis (±2). The primary statistical method employed was exploratory latent class analysis (LCA) using Mplus software (version 8.0). Indicators included dichotomous items from the EPII. The “*Yes, Me*” and “*Yes, Person in Home*” response options were collapsed such that either or both represented a positive item. The “*No*” and “*N/A*” response options were collapsed to represent zero. Items with a base rate of <5% (16 out of 92) were not considered for inclusion as an indicator in the LCA. Among the remaining 76 items, bivariate Spearman correlations were conducted to test for significant associations with the PSS total score. Thirty‐eight items were significantly correlated with the PSS at the 95% confidence level. These items were included in the LCA and spanned each of the thematic domains: Work Life (6 indicators), Home Life (10 indicators), Social Activities and Isolation (5 indicators), Emotional/Physical Health and Infection (14 indicators), and Positive Change (3 indicators).

LCA was applied in several steps. Indicators were entered into the LCA beginning with one class and adding classes incrementally until a unique solution could not be determined with maximum likelihood (ML) methods. Several fit indices were examined and used to determine optimal fit. Information criterion indices include the Bayesian information criteria (Schwartz, 1978), Sample Size Adjusted Bayesian Information Criterion (Sclove, 1987), Consistent Akaike Information Criterion (Bozdogan, 1987), and Approximate Weight of Evidence (Banfield & Raftery, 1993), which are interpreted such that lower values convey better fit. Several relative fit indices were also examined. The Vuong‐Lo‐Mendell‐Rubin Likelihood Ratio Test (Lo et al., 2001) provides comparisons between models, such that nonsignificant values indicate the model with one additional class is not a statistically improved fit over the current model. The Bayes Factor (Wagenmakers, 2007; Wasserman, 2000) is interpreted such that *BF* less than three is considered weak evidence that the model with one fewer class is superior over the model with one additional class, *BF* greater than three but less than 10 conveys moderate evidence, and *BF* greater than 10 conveys strong evidence for the model with one fewer class. The approximate correct model probability (Schwartz, 1978) provides an estimate of the probability that a given model is “correct” among the set of tested models under the assumption that one of the models is “correct”. Entropy values were used to evaluate the quality of classes and ranged from 0 to 1, with values closer to one representing better separation of classes (Ramaswamy et al., 1993). Univariate entropy scores were examined to evaluate the relative contribution of individual items in separate classes.

To examine associations between classes and continuous variables, we used the Mplus DU3STEP procedure described by Vermunt (2010) and Asparouhov and Muthén (2014). Associations between classes and dichotomous variables were examined using the Mplus DCAT procedure described by Lanza et al. (2013). These procedures follow 3‐steps: (1) the LCA is estimated without covariates or distal outcomes, (2) the highest probability of class membership is used to assign classes, and (3) associations between class membership and outcomes are estimated with an adjustment based on classification uncertainty. These methods perform well when class separation is sufficient (i.e., entropy > 0.60). Alpha was adjusted for pairwise comparisons using the Bonferroni procedure.

## 3 RESULTS

### 3.1 EPII base rates and perceived stress

Table 2 presents base‐rates of EPII items and correlations with perceived stress. Sixteen items had a base‐rate ≤5% for either “*Yes, Me*” or “*Yes, Person in Home*”. These included more extreme exposures (e.g., increase in physical conflict with a partner or children in the home, unable to access clean water, unable to access medical care for a serious condition, and living apart from family). Relatively high base‐rate items (>80%) included being separated from family or close friends, having family celebrations canceled or restricted, planned travel or vacations canceled, inability to do enjoyable activities or hobbies, more time sitting down or being sedentary, and in terms of positive change, more appreciative of things usually taken for granted. No items had base‐rates greater than 95%.

**TABLE 2:** EPII base rates, correlation with perceived stress, and univariate entropy scores

		Yes me or other	PSS *r*_s_
*Work/Employment*			
EPII 1	Laid off from job or had to close own business	16.0	−.003
EPII 2	Reduced work hours or furloughed	30.8	−.008
EPII 3	Had to lay‐off or furlough employees or people supervised	7.2	.002
EPII 4	Had to continue to work despite close contact with people who might be infected	34.8	.125**
EPII 5	A lot of time disinfecting home due to close contact with infected people at work	32.4	.103**
EPII 6	Increase in workload or work responsibilities	38.7	.192**
EPII 7	Hard time doing job well because of needing to take care of people in the home	18.4	.171**
EPII 8	Hard time making the transition to working from home	34.5	.179**
EPII 9	Provided direct care to people with the disease	8.7	.063
EPII 10	Provided supportive care to people with the disease	14.1	.097*
EPII 11	Provided care to people who died as a result of the disease^a^	4.8	.107**
EPII 13	Adult unable to go to school or training for weeks or had to withdraw	13.3	.063
*Home life*			
EPII 12	Had a child in home who could not go to school	33.0	.038
EPII 14	Childcare or babysitting unavailable when needed	11.5	.110**
EPII 15	Difficulty taking care of children in the home	12.3	.184**
EPII 16	More conflict with child or harsher in disciplining child or children	13.2	.159**
EPII 17	Had to take over teaching or instructing a child	20.7	.096*
EPII 18	Family or friends had to move into your home	7.5	.032
EPII 19	Had to spend a lot more time taking care of a family member	19.2	.129**
EPII 20	Had to move or relocate^a^	4.6	.082*
EPII 21	Became homeless^a^	0.3	.046
EPII 22	Increase in verbal arguments or conflict with a partner or spouse	20.1	.185**
EPII 23	Increase in physical conflict with a partner or spouse^a^	1.2	.068
EPII 24	Increase in verbal arguments or conflict with other adult(s) in home	13.0	.218**
EPII 25	Increase in physical conflict with other adult(s) in home^a^	0.6	.102**
EPII 26	Increase in physical conflict among children in home^a^	3.1	.107**
EPII 37	Unable to get enough food or healthy food	9.5	.172**
EPII 38	Unable to access clean water^a^	0.5	−.008
EPII 39	Unable to pay important bills like rent or utilities	7.2	.147**
EPII 40	Difficulty getting places due to less access to public transportation or concerns about safety	16.1	.100*
EPII 41	Unable to get needed medications (e.g., prescriptions or over‐the‐counter)	6.1	.045
*Social activities/isolation*			
EPII 27	Separated from family or close friends	90.2	.021
EPII 28	Did not have the ability or resources to talk to family or friends while separated	10.7	−.030
EPII 29	Unable to visit loved one in a care facility (e.g., nursing home, group home)	22.5	−.021
EPII 30	Family celebrations canceled or restricted	90.8	.070
EPII 31	Planned travel or vacations canceled	79.4	.011
EPII 32	Religious or spiritual activities canceled or restricted	51.4	.018
EPII 33	Unable to be with a close family member in critical condition	12.4	.099**
EPII 34	Unable to attend in‐person funeral or religious services for a family member/friend who died	24.2	.043
EPII 35	Unable to participate in social clubs, sports teams, or usual volunteer activities	73.9	−.015
EPII 36	Unable to do enjoyable activities or hobbies	85.1	.080*
EPII 58	Isolated or quarantined due to possible exposure to this disease	30.5	.074
EPII 59	Isolated or quarantined due to symptoms of this disease	10.4	.098*
EPII 60	Isolated due to existing health conditions that increase risk of infection or disease	33.9	−.040
EPII 61	Limited physical closeness with child or loved one due to concerns of infection	44.6	.081*
EPII 62	Moved out or lived away from family due to a high‐risk job^a^	3.5	.061
EPII 63	Close family member not in the home was quarantined	14.9	.073
EPII 64	Family member was unable to return home due to quarantine or travel restrictions	6.4	.027
EPII 65	Entire household was quarantined for a week or longer	13.3	.092*
*Changes in emotional/physical health and infection*			
EPII 42	Increase in child behavioral or emotional problems	16.1	.160**
EPII 43	Increase in child's sleep difficulties or nightmares	12.9	.121**
EPII 44	Increase in mental health problems or symptoms (e.g., mood, anxiety, stress)	69.8	.388**
EPII 45	Increase in sleep problems or poor sleep quality	66.3	.268**
EPII 46	Increase in use of alcohol or substances	32.7	.094*
EPII 47	Unable to access mental health treatment or therapy	12.1	.149**
EPII 48	Not satisfied with changes in mental health treatment or therapy	13.7	.170**
EPII 49	More time on screens/devices (e.g., phone, video games, watching TV)	87.3	.077*
EPII 50	Increase in health problems not related to this disease	21.3	.116**
EPII 51	Less physical activity or exercise	69.8	.104**
EPII 52	Overeating or eating more unhealthy foods (e.g., junk food)	66.7	.113**
EPII 53	More time sitting down or being sedentary	87.6	.070
EPII 54	Important medical procedure canceled (e.g., surgery)	17.3	−.034
EPII 55	Unable to access medical care for a serious condition (e.g., dialysis, chemotherapy)^a^	3.7	.066
EPII 56	Got less medical care than usual (e.g., routine or preventive care appointments)	66.7	.095*
EPII 57	Elderly or disabled family member not in the home unable to get the help they need	9.0	.084*
EPII 66	Currently have symptoms of this disease but have not been tested^a^	2.8	.046
EPII 67	Tested and currently have this disease^a^	0.6	.037
EPII 68	Had symptoms of this disease but never tested	18.1	.096*
EPII 69	Tested positive for this disease but no longer have it^a^	1.4	.025
EPII 70	Got medical treatment due to severe symptoms of this disease^a^	2.6	.082*
EPII 71	Hospital stay due to this disease^a^	0.5	.029
EPII 72	Someone died of this disease while in our home^a^	0.2	.052
EPII 73	Death of close friend or family member from this disease^a^	4.9	.063
*Positive change*			
EPII 74	More quality time with family or friends in person or from a distance	67.2	.091*
EPII 75	More quality time with partner or spouse	51.1	−.039
EPII 76	More quality time with children	36.2	.017
EPII 77	Improved relationships with family or friends	35.7	.069
EPII 78	New connections made with supportive people	22.4	.105**
EPII 79	Increase in exercise or physical activity	27.0	.001
EPII 80	More time in nature or being outdoors	45.2	.025
EPII 81	More time doing enjoyable activities (e.g., reading books, puzzles)	57.4	−.087
EPII 82	Developed new hobbies or activities	28.8	.008
EPII 83	More appreciative of things usually taken for granted	81.3	.051
EPII 84	Paid more attention to personal health	54.3	.023
EPII 85	Paid more attention to preventing physical injuries	41.4	.095*
EPII 86	Ate healthier foods	33.3	.000
EPII 87	Less use of alcohol or substances	12.7	.057
EPII 88	Spent less time on screens or devices outside of work hours	5.5	−.009
EPII 89	Volunteered time to help people in need	19.3	−.009
EPII 90	Donated time or goods to a cause related to this disease	34.0	.045
EPII 91	Found greater meaning in work, employment, or school	33.7	.068
EPII 92	More efficient or productive in work, employment, or school	26.1	−.017

Abbreviation: PSS, Perceived Stress Scale.

^a^ Items with ≤5% base‐rate. Item wording in some cases is abridged.

* *p* < .05

** *p* < .01.

Among the 76 EPII items with ≥5% base‐rate, 38 were significantly positively correlated with the total PSS score, including items from all thematic domains: Work Life (6 indicators; *r*s from .10 to .17), Home Life (10 indicators; *r*s from .04 to .18), Social Activities and Isolation (5 indicators; *r*s from .04 to .05), Emotional/Physical Health and Infection (14 indicators; *r*s from .03 to .18), and Positive Change (3 indicators; *r*s = .03). See Table 2.

### 3.2 LCA fit indices and class solutions

Table 3 presents LCA fit indices. The 5‐class solution was selected as the most parsimonious, best‐fitting model with the smallest BIC, a *BF* conveying strong evidence that the 5‐class model is superior to the 6‐class model, and the largest *cmP* among the tested models, supporting the 5‐class model as the ‘correct’ model. Entropy is 0.868, suggesting a good separation of classes. Average posterior probabilities for ‘most likely class membership’ were high, ranging from 0.90 to 0.99 (see Table 4). Although secondary information criterion fit indices supported a 6‐class model, the BIC is the most commonly used and relied upon fit index for comparing models (Masyn, 2017; Nylund‐Gibson & Choi, 2018) and both the *BF* and *cmP* identified the more parsimonious 5‐class model as superior.

**TABLE 3:** Fit statistics and classification coefficients for latent class analysis

	*d*	LL	BIC	SABIC	CAIC	AWE	VLMR‐LRT *p*	Entropy	*BF*	*cmP*
1 Class	38	−12,117.21	24,480.67	24,360.02	24,379.36	24,398.36	–	–	0.000	.000
2 Classes	77	−11,376.82	23,252.61	23,008.13	23,047.34	23,085.84	<.001	**0.896**	0.000	.000
3 Classes	116	−11,061.00	22,873.93	22,505.64	22,564.45	22,622.70	**<.001**	0.852	0.109	.049
4 Classes	155	−10,912.56	22,829.52	22,337.40	22,416.32	22,493.83	.583	0.864	0.939	.455
5 Classes	194	−10,785.57	**22,828.27**	22,212.32	22,311.10	22,408.10	.180	0.868	**45.241**	.**485**
6 Classes	233	−10,697.33	22,904.51	**22,164.73**	**22,283.38**	**22,399.88**	.443	0.873	832.556	.011
7 Classes	272	**−10,638.31**	23,039.00	22,175.59	22,314.09	22,450.09	.618	0.880	–	.000

Note

Bold values indicate superior fit for each statistic.

Abbreviations: AWE, approximate weight of evidence criterion; BF, Bayes Factor; BIC, Bayesian Information Criterion; CAIC, Consistent Akaike Information Criterion; cmP, correct model probability; *d*, number of parameters; LL, log‐likelihood; *p*, *p*‐value; SABIC, Sample size adjusted BIC; VLRM‐LRT, Vuong‐Lo‐Mendell‐Rubin adjusted likelihood ratio test.

**TABLE 4:** Average posterior probabilities

Classification	Most likely latent class membership				
	1	2	3	4	5
Class 1	**0.985**	0.006	0.002	0.004	0.003
Class 2	0.007	**0.897**	0.059	0.037	0.000
Class 3	0.004	0.037	**0.904**	0.022	0.033
Class 4	0.005	0.021	0.050	**0.903**	0.021
Class 5	0.004	0.000	0.054	0.021	**0.920**

Note

Bolded cells indicate agreement between most likely latent class membership and classification for refined LCAs.

### 3.3 Classes

Table 5 presents univariate entropy scores, with higher values reflecting greater contribution to class separation, and conditional item probabilities across the five classes. Classes 1 and 2 had a relatively greater probability of exposure to stress‐related pandemic experiences across all domains; however, were differentiated such that Class 2 had less exposure to experiences pertaining to childcare and child behavioral health and a somewhat greater probability of exposure to experiences pertaining to adult mental health or alcohol/substance use. In contrast to Classes 1 and 2, Class 3 had relatively low probability of exposure to stress‐related pandemic experiences in the work/employment and home life domains; however, had high probability of adult mental health and sleep problems and unhealthy lifestyle changes. Class 4 had the highest probability of exposure to stress‐related pandemic experiences in the work/employment domain; however, low probability of exposure to experiences in the home life domain. Class 5 was differentiated from all other classes by low probability of exposure to stress‐related experiences across all domains except in the positive change ​domain, which was comparable to other classes. Table 6 presents class differences on sociodemographic characteristics, psychosocial risk, perceived stress and social support, and cumulative counts of experiences across all thematic domains from the full EPII. Conditional item probabilities and class differences on proximal variables were used to further characterize and label the five classes.

**TABLE 5:** 5‐Class solution: conditional item probabilities and univariate entropy scores

	*e*	Classes				
		1	2	3	4	5
*Work/Employment*						
EPII 4. Continue to work despite close contact with people who might be infected	0.169	0.45	0.54	0.06	0.91	0.06
EPII 5. A lot of time disinfecting home due to close contact with infected people at work	0.146	0.42	0.62	0.07	0.75	0.02
EPII 6. Increase in workload or work responsibilities	0.084	0.51	0.65	0.20	0.65	0.13
EPII 7. Hard time doing job well because of needing to take care of people in the home	0.126	0.65	0.28	0.02	0.06	0.05
EPII 8. Hard time making the transition to working from home	0.063	0.54	0.59	0.32	0.25	0.11
*Home life*						
EPII 10. Provided supportive care to people with the disease	0.075	0.14	0.24	0.03	0.39	0.01
EPII 14. Childcare or babysitting unavailable when needed	0.127	0.55	0.01	0.00	0.07	0.01
EPII 15. Difficulty taking care of children in the home	0.168	0.65	0.01	0.00	0.00	0.02
EPII 16. More conflict with child or harsher in disciplining child or children	0.162	0.67	0.03	0.00	0.03	0.01
EPII 17. Had to take over teaching or instructing a child	0.182	0.84	0.01	0.02	0.18	0.08
EPII 19. Had to spend a lot more time taking care of a family member	0.093	0.50	0.43	0.04	0.07	0.08
EPII 22. Increase in verbal arguments or conflict with a partner or spouse	0.067	0.37	0.46	0.14	0.10	0.03
EPII 24. Increase in verbal arguments or conflict with other adult(s) in home	0.067	0.19	0.40	0.10	0.04	0.00
EPII 37. Unable to get enough food or healthy food	0.041	0.17	0.21	0.07	0.04	0.02
EPII 39. Unable to pay important bills like rent or utilities	0.037	0.10	0.19	0.05	0.04	0.02
EPII 40. Lack public transportation	0.043	0.13	0.38	0.17	0.08	0.08
*Social activities and isolation*						
EPII 33. Unable to be with a close family member in critical condition	0.049	0.12	0.37	0.10	0.08	0.02
EPII 36. Unable to do enjoyable activities or hobbies	0.036	0.93	0.94	0.85	0.83	0.73
EPII 59. Isolated or quarantined due to symptoms of this disease	0.041	0.17	0.27	0.07	0.04	0.03
EPII 61. Limited physical closeness with child or loved one due to concerns of infection	0.039	0.34	0.68	0.45	0.45	0.36
EPII 65. Entire household quarantined for a week or longer	0.039	0.15	0.32	0.10	0.09	0.07
*Changes in emotional/physical health and infection*						
EPII 42. Increase in child behavioral or emotional problems	0.154	0.73	0.06	0.04	0.02	0.02
EPII 43. Increase in child's sleep difficulties or nightmares	0.112	0.56	0.04	0.05	0.02	0.01
EPII 44. Increase in mental health problems or symptoms (e.g., mood, anxiety, stress)	0.175	0.88	0.90	0.95	0.57	0.06
EPII 45. Increase in sleep problems or poor sleep quality	0.124	0.76	0.91	0.86	0.53	0.16
EPII 46. Increase in use of alcohol or substances	0.048	0.41	0.47	0.40	0.22	0.11
EPII 47. Unable to access mental health treatment or therapy	0.072	0.14	0.43	0.10	0.00	0.02
EPII 48. Not satisfied with changes in mental health treatment or therapy	0.075	0.17	0.45	0.13	0.01	0.00
EPII 49. More time on screens/devices (e.g., phone, video games, watching TV)	0.038	0.95	0.90	0.92	0.81	0.75
EPII 50. Increase in health problems not related to this disease	0.060	0.22	0.45	0.28	0.05	0.07
EPII 51. Less physical activity or exercise	0.039	0.71	0.87	0.75	0.61	0.55
EPII 52. Overeating or eating more unhealthy foods (e.g., junk food)	0.053	0.81	0.83	0.69	0.62	0.40
EPII 56. Got less medical care than usual (e.g., routine/preventive care appointments)	0.037	0.73	0.80	0.71	0.49	0.61
EPII 57. Elderly/disabled family member not in home unable to get the help they need	0.041	0.14	0.24	0.04	0.06	0.04
EPII 68. Had symptoms of this disease but never tested	0.033	0.21	0.28	0.15	0.25	0.07
*Positive change*						
EPII 74. More quality time with family or friends in person or from a distance	0.033	0.82	0.62	0.64	0.73	0.57
EPII 78. New connections made with supportive people	0.030	0.20	0.36	0.23	0.20	0.15
EPII 85. Paid more attention to preventing physical injuries	0.033	0.36	0.64	0.40	0.36	0.37

Note

*e* = univariate entropy. Darker shades correspond to higher conditional item probabilities. Class 1 “Parents – High Exposure/High Risk (18.1%), Class 2 “Young Adult – High Exposure/High Risk (14.4%), Class 3 “Older Adult – Moderate Exposure/High Risk (31.4%), Class 4 “Young Parents – High Work/Low Risk (17.3%), Class 5 “Older Adult – Low Exposure/Low Risk (18.7%).

**TABLE 6:** Class comparisons on sociodemographic characteristics, psychosocial risk, perceived stress and social support, and cumulative pandemic‐related experiences

	Class 1		Class 2		Class 3		Class 4		Class 5		*χ* ^2^	Class differences
	Prob	*SE*	Prob	*SE*	Prob	*SE*	Prob	*SE*	Prob	*SE*		
*Sociodemographic characteristics*												
Age ≥60 years	.025	0.015	.081	0.048	.312	0.039	.178	0.043	.420	0.057	106.35**	C3,C5 > C1,C2; C5 > C3
Age ≤29 years	.070	0.031	.299	0.074	.160	0.034	.218	0.047	.074	0.039	14.85*	C2,C4 > C1,C5; C3 > C1
Male	.098	0.036	.101	0.049	.198	0.033	.106	0.050	.212	0.042	8.35	
Racial/ethnic minority	.084	0.027	.218	0.050	.056	0.023	.119	0.035	.104	0.033	9.06	
Living with partner	.882	0.034	.659	0.136	.646	0.066	.778	0.047	.729	0.046	24.80**	C1 > C3,C5
Young child in home	.923	0.027	.114	0.054	.089	0.028	.217	0.051	.067	0.026	745.75**	C1 > C2,C3,C4,C5; C4 > C3,C5
Child/adolescent in home	.313	0.045	.027	0.038	.106	0.032	.272	0.055	.058	0.028	37.41**	C1,C4 > C2,C3,C5
Young adult child in home	.131	0.033	.164	0.047	.162	0.032	.298	0.051	.136	0.035	8.02	
Person > 60 in home	.090	0.027	.345	0.126	.380	0.048	.249	0.071	.491	0.062	73.26**	C3,C4,C5 > C1; C5 > C4
Employed	.793	0.039	.658	0.106	.650	0.045	.922	0.036	.662	0.058	38.86**	C4 > C1,C2,C3,C5; C1 > C3
Laid off/ unemployed	.191	0.038	.326	0.100	.154	0.036	.096	0.045	.113	0.040	9.08	
Public or No insurance	.862	0.033	.861	0.056	.794	0.034	.967	0.019	.692	0.057	42.58**	C4 > C1,C3,C5; C1,C2 > C5
Retired	.018	0.013	.024	0.029	.194	0.033	.000	0.000	.221	0.047	74.66	C3 > C1,C2,C4; C5 > C1,C2,C4
Student	.104	0.031	.231	0.064	.095	0.024	.095	0.036	.023	0.017	18.42*	C1,C2,C3 > C5
*Psychosocial risk*												
PC‐PTSD ≥3	.325	0.044	.498	0.086	.277	0.043	.087	0.043	.051	0.022	65.50**	C2 > C3,C4,C5; C1 > C4,C5; C3 > C4,C5
PHQ‐9 ≥ 15	.369	0.048	.645	0.073	.438	0.059	.208	0.052	.009	0.015	189.50**	C2 > C1,C3,C4,C5; C1 > C4,C5; C3 > C4,C5; C4 > C5
GAD‐7 ≤ 15	.451	0.050	.513	0.092	.382	0.050	.183	0.059	.000	0.000	214.86**	C2 > C4,C5; C1 > C4,C5; C3 > C4,C5; C4 > C5

	Class 1		Class 2		Class 3		Class 4		Class 5		*χ^2^*	Class differences
	*M*	*SE*	*M*	*SE*	*M*	*SE*	*M*	*SE*	*M*	*SE*		
*Perceived stress and social support*												
PSS	23.56	0.33	23.93	0.50	22.54	0.27	21.74	0.44	19.04	0.31	135.05**	C1,C2 > C3,C4,C5; C3,C4 > C5
Social support	20.15	0.43	18.41	0.63	19.20	0.45	23.37	0.34	21.64	0.59	91.13**	C4 > C1,C2,C3,C5; C5 > C1,C2,C3
*EPII domain totals*												
Work life	3.66	0.19	4.42	0.21	1.25	0.09	4.09	0.15	0.50	0.09	832.67**	C2 > C1,C3,C5; C4 > C3,C5; C3 > C5
Home life	5.68	0.19	3.29	0.22	1.03	0.10	1.27	0.15	0.32	0.07	834.73**	C1 > C2,C3,C4,C5; C2 > C3,C4,C5; C3,C4 > C5
Social/Isolation	6.94	0.69	10.14	0.50	6.48	0.17	6.31	0.31	5.90	0.20	128.95**	C2 > C1,C3,C5; C3 > C5;
Emotional/Physical	8.70	0.25	9.70	0.25	7.29	0.11	5.28	0.18	3.78	0.16	643.70**	C2 > C1,C3,C4,C5; C1 > C3,C4,C5; C3 > C4,C5; C4 > C5
Positive	7.61	0.32	8.14	0.41	6.54	0.24	7.74	0.40	6.28	0.35	22.11**	C2 > C3,C5; C1 > C3,C5; C4 > C3,C5

Note

Class difference represent pairwise comparisons that are significant at *p* < .005 (Bonferroni adjustment).

* *p* < .01

** *p* < .001.

#### 3.3.1 Class 1 “Parents – high exposure/high risk”

This class represents about a fifth of the sample and is characterized by a greater probability of living with a partner and caring for a child in the home. Individuals in this class were less likely to report caring for an older adult in the home. This class was differentiated from Classes 4 and 5 by a having greater probability of reporting cumulative pandemic‐related experiences in the home life and emotional/physical health domains. Specifically, individuals in this class were relatively more likely to report needing to continue to work despite the risk, experiencing childcare issues, having to take over teaching at home, using harsher discipline, observing an increase in child behavior problems, and experiencing an increase in verbal conflict with a partner. This class was also differentiated from Classes 4 and 5 by having a greater probability of screening positive for possible PTSD, depression, and anxiety and reporting higher levels of perceived stress, but also from Classes 3 and 5 in reporting a higher level of positive experiences related to the pandemic.

#### 3.3.2 Class 2 “Young adult – high exposure/high risk”

This class represents about 14% of the sample and is characterized by a relatively greater probability of being a young adult. This class was also differentiated from Classes 4 and 5 by having a higher probability of cumulative pandemic‐related experiences in the work, home life, social/isolation, and emotional/physical health domains. Specifically, individuals in this class were relatively more likely to report continuing to work despite the risk, having a hard time doing their job well, spending significant time caring for a family member, increased verbal conflict with a partner or other adult in the home, barriers to public transportation and obtaining healthy food, limited closeness with a loved one, barriers to mental health treatment, and dissatisfaction with changes in mental health care. This class also was differentiated from Classes 4 and 5 by having a higher probability of screening positive for PTSD, depression, and anxiety, and reporting higher levels of perceived stress. Relative to other classes, Class 2 also had a higher probability of reporting positive experiences associated with the pandemic than Classes 3 and 5.

#### 3.3.3 Class 3 “Older adult – moderate exposure/high risk”

This class is the largest class representing about 30% of the sample. Class 3 was differentiated from other classes by having a higher probability of being over the age of 60 and being retired. This class also had a relatively lower probability of cumulative pandemic‐related experiences in the work, home life, and social/isolation domains, but a relatively higher probability of experiences in the emotional/physical health domain, similar to Classes 1 and 2 but with lower probability of reporting barriers to accessibility of mental health treatment. This class was differentiated from Classes 4 and 5 by having a higher probability of screening positive for PTSD, depression, and anxiety, and reporting higher levels of perceived stress, and from Classes 1, 2, and 4 in reporting fewer positive pandemic‐related experiences.

#### 3.3.4 Class 4 “Young parents – high work/low risk”

This class represents about 17% of the sample. Individuals in this class had a high probability of being a young adult and caring for a child in the home. Individuals in this class were also relatively more likely to be employed, but also to have public or no insurance. This class had a relatively high probability of reporting cumulative pandemic‐related experiences in the work domain, but a relatively low probability of experiences in the home life and social/isolation domains. This class was differentiated from Classes 1, 2, and 3 by having a lower probability of screening positive for PTSD, depression, or anxiety, reporting lower levels of perceived stress, and reporting higher levels of social support, and from Classes 3 and 5 in reporting a higher number of positive pandemic‐related experiences.

#### 3.3.5 Class 5 “Older adult – low exposure/low risk”

This class represents about 19% of the sample and was differentiated from Classes 1, 2, and 4 by having a higher probability of being an older adult and being retired. Individuals in this class were least likely, relative to other classes, to report pandemic‐related experiences in all domains. This class was differentiated from Classes 1, 2, and 3 by having a lower probability of screening positive for PTSD, depression, or anxiety, reporting lower levels of perceived stress, and reporting higher levels of social support, and from Classes 1, 2, and 4 in reporting fewer positive experiences.

#### 3.3.6 Summary of Class Differences

Overall, classes with the most negative pandemic‐related experiences included Classes 1, 2, and 3. These classes also tended to include a greater proportion of individuals screening positive for PTSD, depression, and anxiety, reporting higher levels of perceived stress, and reporting lower levels of social support. Additionally, two of those classes that had a higher probability of younger or mid‐life adults (Classes 1 and 2) along with the class with a high probability of young adults (Class 4) reported more positive pandemic‐related experiences than the two classes that had a higher probability of older adults (Classes 3 and 5).

## 4 DISCUSSION

The current findings demonstrate the utility of the EPII in identifying unique profiles (classes) based on patterns of stress‐related pandemic experiences using a person‐centered analytic approach. Five unique profiles were identified as the best‐fitting solution. Findings also provide evidence of differential associations between identified profiles and sociodemographic characteristics and psychosocial functioning.

Caregivers of children and adolescents were more likely to be classified into two profiles differentiated by exposure level and psychosocial risk. Individuals in the “*Parents – High Exposure/High Risk*” (Class 1) profile comprised about a fifth of the sample and were likely to report cumulative pandemic‐related experiences. This profile was specifically differentiated from other profiles by having a greater probability of caring for a young child and reporting difficulties with childcare and teaching at home, increased child emotional and behavioral problems, and perhaps consequently, increased use of harsh discipline. In contrast, individuals classified in the “*Young Parents – High Work/Low Risk*” profile (Class 4, 17%) were more likely to be a young adult with both children and an older adult in their household and to report cumulative stressful experiences in the work domain and relatively fewer adverse experiences in the home life and social/isolation domains, and fewer child emotional or behavioral problems. These two profiles both included a high number of positive pandemic‐related experiences but differed on indicators of psychosocial risk, with individuals with the “*Parents – High Exposure/High Risk*” profile more likely to report high perceived stress and to screen positive for possible PTSD, depression, and anxiety, and less likely to report high levels of social support relative to individuals classified in the “*Young Parents – High Work/Low Risk*” profile.

The two ‘caregiver’ profiles tell different narratives of family experiences over the pandemic, with one profile clearly set apart by a heavier burden including caring for young children, fewer social resources, and greater risk for psychosocial impairment. Although the current study did not collect information about child functioning, the psychosocial risk of the “*Parents – High Exposure/High Risk*” profile likely extends to children in the home. For example, a recent study using the EPII demonstrated that parents’ emotional availability and ability to maintain a stable home routine served to buffer the impact of pandemic‐related stress on children's emotional and behavioral problems (Cohodes et al., 2021). Membership in the high‐risk class might indicate a need for family support programs and services to assist with parent management and home education.

Older adults were more likely to be classified in two profiles that were differentiated by exposure level and psychosocial risk. Individuals classified in the “*Older Adult – Low Exposure/Low Risk*” profile represented about a fifth of the full sample and were likely to be retired and to report relatively fewer pandemic‐related experiences across thematic domains. Despite their age difference, Individuals with this older adult profile were similar to those classified in the “*Young Parents – High Work/Low Risk*” profile in that they tended to report the lowest levels of perceived stress and the highest social support, while also less likely to screen positive for PTSD, depression, or anxiety. In contrast, individuals classified in the “*Older Adult – Moderate Exposure/High Risk*” profile represented the largest proportion of the sample (30%) and were more likely than other older adults to report cumulative pandemic‐related adverse experiences in the work, home life, and emotional and physical health domains, with a high probability of reporting increased social isolation, mental health, sleep, and alcohol/substance use problems, as well as increased negative lifestyle behaviors (e.g., less physical activity, unhealthy eating). Like the other high‐risk profiles, individuals with this profile were likely to report high levels of perceived stress and to screen positive for PTSD, depression, and anxiety. Both the lower risk and higher risk older adult classes were notably less likely to report positive pandemic‐related experiences than the young or mid‐life adult classes.

The physical, psychological, and social vulnerabilities that come with older age may make managing life with COVID particularly challenging for older individuals and lead to compound risk for psychosocial impairment (Banerjee, 2020). With an increased risk of COVID infection and poor prognosis following infection, older individuals have had to take serious precautions, in some cases having to forgo routine or necessary medical care or discontinue any social activity. Further, cognitive impairment and physical disabilities, combined with disruptions in services, have made COVID especially burdensome for some older adults. Access to supportive resources and positive life experiences may be particularly diminished in the pandemic for older adults. The two profiles representative of older adults in the current study reflects two distinct narratives of pandemic life – one in which there may be insufficient resources, increased social isolation, and high risk for adverse outcomes, and one in which there may be safeguards and resources in place to help older individuals adapt to increased restrictions, lifestyle changes, and disruptions in services and to remain socially connected despite social distancing measures. These patterns might suggest the need for intensive efforts to help older individuals adapt to the many changes to their daily activities and implement strategies for risk prevention while still maintaining critical social connections.

Finally, the “*Young Adult – High Exposure/High Risk*” profile representing about 14% of the sample and consisting predominately of younger adults had a high probability of adverse pandemic‐related experiences across all domains, including having to work despite increased risk of infection, needing to spend significant time caring for a family member, increased verbal conflict with a partner or other adult in the home, limited closeness with a loved one, and barriers to mental health treatment. Individuals classified in this profile had the highest likelihood of screening positive for PTSD, depression, or anxiety. These individuals may include “essential” workers in healthcare, social and environmental services, and commercial settings suggested to be a heightened risk for psychosocial impairment due to work‐related stress (Kang et al., 2020). These individuals may also include those with preexisting mental health difficulties or life stressors that may have exacerbated the impact of the pandemic on emotional health (Mukhtar & Rana, 2020). This profile highlights the need for specialized services geared toward serving the highest risk individuals and those that might need assistance in multiple life domains, including work, family life, and personal health.

The current study has limitations. Notably, the sample represents a convenience sample recruited through social media advertisements, listservs, and utilization of a research participant database. As such, the sociodemographic composition of the sample is quite homogenous and not representative of lower income and racially/ethnically diverse populations. It is quite possible that experiences with low base‐rates in the current sample may have been more prevalent in a sample representative of a more diverse, under‐resourced population. Despite this important limitation, the value of this preliminary study lies in its capacity to substantiate the EPII’s ability to identify meaningful profiles of pandemic‐related experiences with linkages to indicators of perceived stress and impairment, even in a non‐diverse sample. Future research will need to extend these findings to more diverse populations.

Another consideration is that rates of pandemic‐related experiences on the EPII may change over time. The current study surveyed individuals during and after peak rates of COVID‐19 in the Northeast region. It is possible that response patterns on the EPII may have been different if the sample was surveyed later, as more time passed provides greater opportunity for these experiences to occur. Ideally, the EPII would be administered at multiple time points across the pandemic so as to track changes in rates of different types of experiences.

In summary, the current study represents the first investigation to use a person‐centered, data‐driven approach to characterizing and contextualizing negative and positive experiences of individuals during an unprecedented pandemic crisis. It serves to provide an example of an approach that may be suitable for studying future disasters and public health emergencies, as well as support a novel instrument for measuring tangible, pandemic‐related experiences. More research will be necessary to understand how pandemics such as COVID‐19 impact personal and social experiences over time and how patterns and impacts might differ in various populations.

## CONFLICT OF INTERESTS

None declared.

### PEER REVIEW

The peer review history for this article is available at https://publons.com/publon/10.1002/brb3.2197.

## Data Availability

Research data are not shared.
